# Supplementary material for: A qualitative examination of the experiences and perspectives of interprofessional primary health care teams in the distribution of the COVID-19 vaccination in Ontario, Canada
Source: PLoS One. 2024 Jun 10;19(6):e0304616. doi: 10.1371/journal.pone.0304616 (PMC11164373; doi:10.1371/journal.pone.0304616)
Supplement: Attachment — Submitted filename: Comment Reviewer A Qualitative Examination of the Facilitators and Barriers Shaping Primary Health Care.pdf [file pone.0304616.s002.pdf]

A Qualitative Examination of the Facilitators and Barriers Shaping Primary Health Care Teams' Experiences in the Distribution of the COVID-19 Vaccination in Ontario, Canada

Comments to the Author

Congratulations on the submitted manuscript. The topic is timely and will be of interest to the readers of the journal. However, a few changes are suggested to improve the clarity of this manuscript. I have several recommendations and questions about the manuscript.

No	Page	Descriptions of item	Comment
1.		**Abstract**<br>A descriptive qualitative design informed this study. We conducted focus groups with a range of interprofessional healthcare providers, administrators, and staff working in PHC teams across Ontario. Eight focus groups were conducted with 39 participants representing the six health regions of the province.	Harmonize the statements:<br>A qualitative approach was used for this study, which involved 39 participants from six health regions of the province. Eight focus groups were conducted----
2.		**Introduction**	Very good explanation.
3.	11	**Methodology**<br><br>Our study used a qualitative description approach, using focus groups to explore the experiences of participants.<br><br>Data collection and data analysis took place simultaneously, with data analysis following the thematic analysis process as outlined by Braun & Clark [29].	Here need to explain how many participants were involved in this study.<br><br>Explain the steps of data analysis using Braun & Clark(2006), to make it more clear for the readers.
4.		**Discussion**	Very good.
		**References**	Most of the citations are above 5 years. Very good.
